# Would you like to be contacted about future research?

**DOI:** 10.1186/s13104-021-05884-2

**Published:** 2021-12-20

**Authors:** Lisa Newington, Caroline M. Alexander, Mary Wells

**Affiliations:** 1 Imperial College Healthcare NHS Trust, London, UK; 2 Faculty of Medicine, Imperial College London, London, UK

**Keywords:** Research recruitment, Research participation, Research gatekeepers, Research consent, Research management

## Abstract

Many research participants are willing to be contacted about future research opportunities, however this question is not always asked. Furthermore, if participants do consent for contact about future research, this information is not always accessible to other research teams. We discuss our experience of recruiting individuals who have previously taken part in healthcare research and suggest potential strategies to support this process and enable greater research participation.

## Introduction

We are currently conducting a qualitative interview study to explore participants’ perceptions of research impact [1]. Interviewees are not limited to participants from a particular research study or programme of research, instead, the eligibility criteria are broad and encompass involvement in healthcare research within the past three years. This could be at any UK site and includes both research participants and patient advisory group members. Recruitment involves establishing a series of Patient Identification Centres (PICs) who will send study invitations to their past/present participants and patient advisors, where consent has been provided for contact about future research. Interested individuals are invited to contact us directly, preventing any issues with data sharing.

At face value, this appears to be a simple recruitment strategy, however, we have encountered several obstacles in accessing potential participants: (1) participants were not asked if they consent to contact about future research; (2) if asked, the data were not stored in a way that enabled rapid filtering and identification of those who agreed; (3) archived research datasets were not accessible; and finally; (4) there was no systematic method of identifying all potential sources of participants.

We would like to share our reflections on this experience and discuss potential strategies to resolve these issues and enable greater research collaboration and participation.

## Main text

### Consent for future contact—we all need to ask the question

Our preliminary search for potential PIC locations uncovered that many researchers do not include a question on their study consent forms asking their participants if they would like to be contacted about future research opportunities. By not asking the question, these research-engaged participants are completely inaccessible to other researchers, or even to future studies run by the same research team. Our own organisation was, until recently, guilty of this omission, and personal communication with researchers from a range of institutions suggests that template consent forms do not consistently include a question about contact regarding future research. Importantly, this question is not included in current Medical Research Council and Health Research Association consent form guidance or templates [2], which leaves the onus on individual researchers to remember to add this crucial question to their consent forms.

Other strategies to enable individuals to be contacted about future research opportunities include generic consent processes, such as the ‘Research for the Future’ community in North West England [3]. This scheme enables individuals to sign-up directly if they agree to be contacted about relevant research in the future. In addition, NHS staff can recruit patients during standard healthcare visits. This initiative has been successful; more than 1500 participants were recruited to 33 studies during a single year (2016–17), and the team won a digital design award [4]. Similar registries are being using in other UK locations, for example Voice, which was established at Newcastle University in affiliation with the UK National Innovation Centre for Ageing [5]; SHARE, the Scottish Health Research Register and Biobank [6]; and the North West London Health Research Register [7]. Integrated registries containing the health, employment, and demographic information of all citizens are well established in Sweden, Denmark, Finland and Norway. With appropriate permissions, data from these registries can be used for health and social care research without expressed consent [8]. Other strategies include the use of confidentiality waivers to aid the identification and recruitment of potential research participants. In one case, NHS Trusts were able to send details of patients who met the broad research eligibility criteria directly to a research team [9]. The criteria were a specific ICD-10 code and certain demographic criteria, which were easily screened via the electronic health records system, and allowed the research team to personally contact eligible patients. Similar consent waivers are discussed in the Australian National Statement of Ethical Conduct in Human Research (chapter 2.3), along with opt-out research consent processes [10], although it is not clear how commonly these approaches are used. The latter approach is also adopted elsewhere, and assumes that everyone is willing to be informed about relevant healthcare research unless they have ‘opted-out’ at either the local [11] or national level [12].

While these proactive strategies do not appear to be the norm, and do not specifically address the issue of future contact for existing research participants, they do highlight potential strategies to aid prospective identification of potential participants.

### Identifying those who agreed to be contacted

If participants have agreed to be contacted about future research, this needs to be recorded in a secure and accessible way that enables these individuals to be easily identified and contacted. This requires the consent information to be completed (or at least duplicate entered) electronically to enable screening for those who agreed to future contact. There are obvious ethical and research governance issues around the incorrect contact of individuals who have not given consent, and clear processes are required to monitor and prevent this from occurring. This needs suitable IT infrastructure, and support for researchers to develop appropriate database management skills. We encourage researchers at all levels to think about future-proofing their research databases at setup, and encourage Research and Development teams to facilitate this by providing appropriate training and support.

### Finding the gatekeepers for archived data

Our current study focuses on the views of people who took part in healthcare research within the past three years. Several clinical academics we approached had led a study as part of a doctoral or postdoctoral fellowship but now worked at a different organisation. Their study was complete and the data archived, but it was not clear who was now responsible for granting access to the data, or who would be able to perform a screening search and send out study invitations. The latter two activities often need to be completed by the original study team as part of the original ethics approval process.

Discussions around gatekeeping in research tend to focus on access to participants for initial recruitment to a study [13, 14], but these issues also relate to access to those who have consented to future contact, and this warrants further consideration. Importantly, this is not just a recruitment issue for research teams, it also raises ethical questions around access to research and data storage. For example, what if barriers to re-contact prevent individuals from accessing research interventions that may deliver clinical or other benefits? Who is responsible for ensuring access if individuals have provided consent for future contact? How long should participant’s details be held before they are asked to re-consent?

### Identifying sources of potential participants

The final issue we encountered was identifying sources of potentially eligible participants for our study. Our research question was broad and related to participation in all types of research within the spectrum of clinical areas. We used our personal networks, social media, conference presentations and snowballing to spread the word, but it’s still likely that we have missed many potential recruitment sites. Centralised databases of research participants, for example UK Biobank [15], or databases of routinely collected data, for example Electronic Health Records Systems [16, 17], play important contributions to research recruitment. A central database of research that has been approved through the national research ethics process is an important starting point. In the UK, the NHS Health Research Authority website has a ‘research summaries’ function which allows the database of submitted studies to be searched by key word, broad type of study (research, database or tissue bank) and date of application [18]. However, the outputs are not easy to separate into clinical area or type of research (for example trials, observational, qualitative), and the search function would benefit from additional filtering criteria to aid the identification of relevant studies. Similarly, national and international trials registries contain information about ongoing and completed randomised controlled trials, but there is no universally adopted system for other types of research.

## Conclusion

Fortunately, the issues we identified were not insurmountable. This was largely due to the support of individual researchers and research teams who were willing to aid us on our quest. We have managed to open PIC locations across the country, enabling our study invitation to be sent to individuals who have previously taken part in healthcare research. However, there were lots of dead-ends and the process differed at every site. There will be many individuals who agreed to be contacted about future research, but for one or more of the reasons discussed above, were unable to be identified. We see this as a contribution to research waste. Elements of research waste include: failure to ask a research question that is meaningful to patients or other stakeholders; poor study design and conduct; unnecessary repetition of existing research; or failure to complete or publish a study [19]. We believe that giving individuals the option to be contacted about future research, and having mechanisms in place to facilitate the identification of these individuals, are important aspects of study design and conduct, and thereby minimise future research waste.

The current NHS constitution includes a pledge “to inform you of research studies in which you may be eligible to participate” [20], but this is difficult to fully operationalise, as illustrated by our study. The current process requires coordination by individual research teams as part of an ‘opt-in’ system. An alternative strategy is to switch the assumption of consent for contact (or re-contact) to one of ‘opt-out’. It has been suggested that opt-out approaches increase patient access to research opportunities and create study samples that are more representative of the general population [11], however the impacts for those who opt-out also need to be considered [12].

## Outlook

As a healthcare research community, we need to ensure that research participants are given the option to consent to be contacted about future research. We also need clear local systems and governance to enable screening and access to those individuals who agreed to be contacted. Several innovative databases exist where individuals can prospectively register and agree to be contacted about relevant research opportunities, generally without previous research participation. A similar approach for participants who have been recruited through existing research studies may aid recruitment, while also reducing research time and resources. We welcome further discussion on these issues.

## Data Availability

Data sharing not applicable to this article as no datasets were generated or analysed for this commentary.

## Abbreviations

HRA: Health Research Authority (UK)
ICD-10: International Statistical Classification of Diseases and Related Health Problems 10th Revision
NHS: National Health Service (UK)
PIC: Participant identification centre
REC: Research Ethics Committee (UK)
